# Advanced Heart Failure: Therapeutic Options and Challenges in the Evolving Field of Left Ventricular Assist Devices

**DOI:** 10.3390/jcdd11020061

**Published:** 2024-02-16

**Authors:** Michael Antonopoulos, Michael J. Bonios, Stavros Dimopoulos, Evangelos Leontiadis, Aggeliki Gouziouta, Nektarios Kogerakis, Antigone Koliopoulou, Dimitris Elaiopoulos, Ioannis Vlahodimitris, Maria Chronaki, Themistocles Chamogeorgakis, Stavros G. Drakos, Stamatis Adamopoulos

**Affiliations:** 1 Heart Failure, Transplant and Mechanical Circulatory Support Units, Onassis Cardiac Surgery Center, 17674 Athens, Greece; antonopoulos.m@hotmail.com (M.A.); bo_mic@yahoo.com (M.J.B.); evanleont@gmail.com (E.L.); nkoger@gmail.com (N.K.); koliopoulou.a@gmail.com (A.K.); yiannisvlahodimitris@yahoo.gr (I.V.);; 2 Cardiac Surgery Intensive Care Unit, Onassis Cardiac Surgery Center, 17674 Athens, Greece; delaiopoulos@gmail.com (D.E.);; 3 Division of Cardiovascular Medicine, University of Utah School of Medicine, Salt Lake City, UT 84112, USA

**Keywords:** advanced heart failure, left ventricular assist devices, mechanical circulatory support

## Abstract

Heart Failure is a chronic and progressively deteriorating syndrome that has reached epidemic proportions worldwide. Improved outcomes have been achieved with novel drugs and devices. However, the number of patients refractory to conventional medical therapy is growing. These advanced heart failure patients suffer from severe symptoms and frequent hospitalizations and have a dismal prognosis, with a significant socioeconomic burden in health care systems. Patients in this group may be eligible for advanced heart failure therapies, including heart transplantation and chronic mechanical circulatory support with left ventricular assist devices (LVADs). Heart transplantation remains the treatment of choice for eligible candidates, but the number of transplants worldwide has reached a plateau and is limited by the shortage of donor organs and prolonged wait times. Therefore, LVADs have emerged as an effective and durable form of therapy, and they are currently being used as a bridge to heart transplant, destination lifetime therapy, and cardiac recovery in selected patients. Although this field is evolving rapidly, LVADs are not free of complications, making appropriate patient selection and management by experienced centers imperative for successful therapy. Here, we review current LVAD technology, indications for durable MCS therapy, and strategies for timely referral to advanced heart failure centers before irreversible end-organ abnormalities.

## 1. Advanced Heart Failure (ADHF)

Heart Failure (HF) is a complex clinical syndrome with high morbidity and mortality, imposing a significant burden on healthcare systems in terms of resources and costs [1]. HF incidence in the adult population is 1–2%, but it reaches up to 10% in people >70 years old. Although incidence has remained stable over the years, HF prevalence is increasing due to the aging population and better survival following acute myocardial infarction [2]. Improved outcomes have been achieved with new pharmacological treatments and device (ICD/CRT) therapy. However, nearly 10% of HF patients will progress to ADHF. These patients experience poor quality of life, recurrent hospitalizations, and 25–50% mortality within 1 year [3,4,5]. The Heart Failure Association of the ESC has updated the criteria for the definition of ADHF [6] (Table 1).

For ADHF patients with advanced age (>75 years) and/or severe co-morbidities, palliative care is the therapy of choice [7]. The PAL-HF trial was the first randomized controlled trial to show clinical benefit with improvement in quality of life measures by using an interdisciplinary intervention in end-stage HF patients [8]. For the rest of the patients, we should consider advanced therapies, including heart transplantation (HTx) and durable mechanical circulatory support (MCS). HTx, with recent 1- and 10-year survival rates of approximately 90% and 60%, respectively, is the treatment of choice regarding longevity and quality of life in selected patients with ADHF [9]. However, shortage of donor organs, prolonged waiting-list time, and patient co-morbidities remain significant limitations, making HTx a treatment available to only a small proportion of patients with ADHF [10]. LVADs have emerged as an effective and viable form of therapy for this patient group [11]. Though this field is experiencing explosive growth, LVAD recipients are at risk for serious adverse events, making appropriate candidate selection the key to optimal treatment outcomes.

## 2. Anatomy of the Current LVAD Technology

According to the INTERMACS registry in the USA, 95% of implanted LVADs in the last decade are Continuous Flow (CF-LVADs) or non-pulsatile LVADs [12].Those CF-LVADs have been technically upgraded and have better outcomes compared to the first-generation pulsatile devices [13,14].Overall survival of patients after LVAD implantation (either as a bridge to transplant or as destination therapy) is 83% at 1 year and 73% at 2 years [15]. First-generation devices were bulky, pneumatically driven, and pulsatile, leading to high rates of morbidity, mortality, and device failure, thus significantly limiting the duration of support [16]. New-generation CF-LVADs have smaller sizes, are easier to implant, and have a decreased risk of infection [17]. Their size currently permits implantation via a minimally invasive approach [18]. Moreover, placement in patients with smaller bodies or left ventricular sizes and even right-sided support became feasible [19,20,21]. CF-LVADs have also demonstrated improved durability (>10 years in some cases), they produce less noise and they are user-friendly [22,23]. Furthermore, the cost effectiveness of LVAD therapy is continuously improving, irrespective of the pre-implant strategy (bridge to transplant or destination therapy), especially with new-generation devices, and this can promote LVAD utilization in more patients and in weaker healthcare systems [24]. Due to the continuous flow, most patients have undetectable peripheral pulses. It should be mentioned that CF-LVADs have been associated with gastrointestinal bleeding, arterial-venous malformations, aortic insufficiency, and a lower rate of left ventricular recovery, partially explained by the unique continuous flow physiology [25].

**Figure 1:**
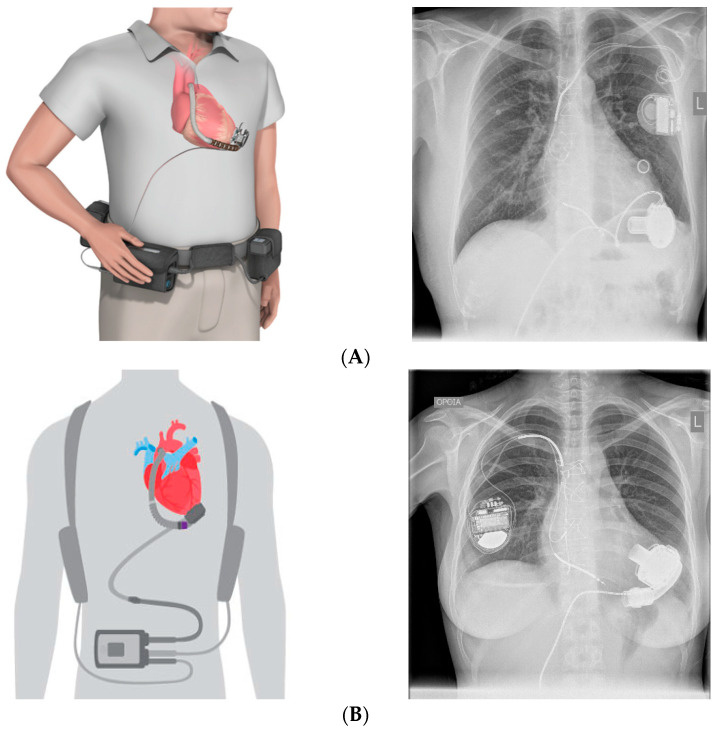
Third-generation CF-LVADs, schematic demonstration, and chest X-ray appearance from patients in our unit. (**A**) HeartWare HVAD™ (Medtronic), (**B**) HeartMate 3™ (Abbott).

The most frequently implanted CF-LVADs are third-generation, intrapericardial, magnetically levitated, centrifugal pumps, like HeartWare™ HVAD™ (Medtronic, Minneapolis, MN, USA) and the more recently approved fully magnetically levitated HeartMate 3™ (Abbott Laboratories, Abbott Park, Illinois, US) (Figure 1). However, on June 2021, Medtronic announced the withdrawal of the HVAD from the global market due to the risk of neurological adverse events, mortality, and potential failure to restart [26].

Regardless of their design, the pumps draw blood from the apex of the left ventricle through an inlet cannula (inflow) and advance it via an outlet cannula (outflow) into the ascending aorta. The pump is connected to the external control system (controller) with a percutaneous cable (driveline), which exits the body from the abdominal wall. The controller monitors the pump function and displays the revolutions per minute (rpm), flow (L/min), and power (Watts) so adjustments to LVAD speed can be made according to the clinical setting. The pump is supplied with energy via portable rechargeable batteries and/or an AC adaptor. Each device has specific external equipment to allow the patient to move around freely.

**Figure 2:**
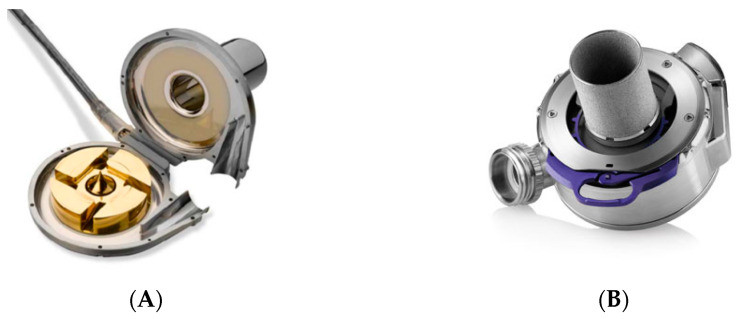
Pumps of the most frequently implanted CF-LVADs. (**A**) Mixed magnetic and hydrodynamic levitation, HeartWare™ HVAD™ (Medtronic), withdrawn from the market (**B**) full magnetic levitation, HeartMate 3™ (Abbott), currently the only FDA-approved CF-LVAD.

Third-generation CF-LVADs have centrifugal pumps that direct flow perpendicular to the axis of rotation. The pump houses the impeller, a spinning disk that is magnetically levitated and can produce up to 10 L of blood flow per minute. These novel devices have no mechanical bearings and allow wide passages for the bloodstream in order to reduce mechanical stress and thrombus formation (Figure 2). Among the existing CF-LVADs, HeartMate3™ is fully magnetically levitated and periodically changes rotor speed to create intrinsic pulsatility. In the 2-year outcomes of the MOMENTUM 3 trial, HeartMate 3™ was superior to HeartMate II™ in improving survival free from disabling stroke and the need to remove or replace the pump. There was a significant reduction in pump thrombosis, which occurred only in 1.1% of the patients in the centrifugal pump group compared to 15.7% in the axial flow pump group. Remarkable was also the overall survival at 2 years (82.8%) in the centrifugal flow arm, which approximates the survival of HTx [27].

New technological advances, like fully implantable LVADs with transcutaneous energy transmission, are being awaited in the near future. The first human experience with this innovative technology, using a coplanar energy transfer (CET) system coupled with a CF-LVAD, has already been reported [28].

## 3. Indications for Durable MCS

The initial approach to an LVAD candidate should be the evaluation for eligibility criteria for HTx after meticulous assessment by an experienced heart team. Although many similarities exist in the selection criteria between the two advanced therapies, significant differences must be considered in order to select the appropriate candidates. High pulmonary vascular resistance or a recently treated malignancy (without metastases) are absolute contraindications for HTx. In contrast, the criteria for MCS are more liberal. On the other hand, some patients could experience good outcomes with HTx compared to MCS with an LVAD. This applies to patients with unfavorable anatomy (small and/or hypertrophic left ventricle) or severe right ventricular dysfunction, which may be further aggravated by LVAD implantation, leading to irreversible right ventricular failure [6,29,30,31].

Four major indications for LVAD implantation currently exist: (1) bridge to transplantation (BTT), (2) destination therapy (DT), (3) bridge to decision (BTD), and (4) bridge to recovery (BTR).

Bridge to transplantation refers to patients who are eligible candidates for HTx but are not able to maintain adequate organ perfusion despite treatment with inotropes and temporary MCS. Because of hemodynamic instability and increased risk of mortality, patients are too sick to wait for a potential donor and require durable MCS. Within this context, the LVAD improves survival (88% at 1 year) and quality of life, and the patient is presented for transplant in a compensated state with improved renal, hepatic function, and nutritional status [15,32]. One out of 2 transplanted patients is already supported mechanically at the time of HTx, whereas 30% of listed LVAD patients undergo HTx within the first year of support [9,33]. Complications after LVAD implantation are a major concern, and as a consequence, after 24 months of support, the implant strategy of 25–30% of patients may change from BTT to DT strategy [34]. Infections and stroke are major complications that can force an LVAD recipient to become ineligible for HTx [30,31]. Furthermore, allosensitization during LVAD support is not infrequent but represents a risk factor either for delaying HTx and/or for antibody-mediated rejection after HTx [35]. Finally, the risks of a second cardiac surgery, the ones related to the expected HTx, are also a disadvantage of the BTT strategy [31]. However, the post-transplant survival of patients on LVAD support before HTx is not significantly different from those who did not require LVAD as a BTT. Additionally, the duration of MCS does not negatively affect survival after HTx [36].

Destination therapy refers to the implantation of a permanent LVAD in patients who are not eligible for HTx. Due to the growing population of patients with ADHF, the stagnant number of donor organs, and the increased durability of the newer devices, the DT strategy is growing. DT accounts for nearly 50% of the implants in the current era and is the leading indication for LVAD therapy in some countries [15,37].The landmark REMATCH trial was the first to compare the efficacy of LVAD therapy versus optimal conservative treatment in patients with end-stage HF who were ineligible for HTx. The device used in the study was the first-generation pulsatile HeartMate XVE LVAD. The study was completed in 2001 and showed a 48% risk reduction in mortality in the LVAD group, thus providing the indication for DT. 1-year survival in patients receiving MCS was 52% vs. 25% in those who received medication, while the 2-year survival was 23% and 8%, respectively. In addition to survival benefits, the group of patients treated with LVAD had improved quality of life, although the risk of experiencing severe complications was doubled compared to the conservative treatment arm [38]. Subsequently, the multicenter HeartMate II trial randomized 200 transplant-ineligible patients to receive therapy with the HeartMate XVE vs. second-generation HeartMate II LVAD. This study showed dramatically better event-free survival with the second-generation CF-LVAD. One-year survival was 68% (HM II) vs. 55% (HM XVE), while 2-year survival was 58% and 24%, respectively [39]. A remarkable further improvement in 1- (80%) and 2-year (69%) survival has been recently demonstrated with the DT approach, in parallel with the increasing rate of implantation of the third-generation CF-LVADs [15].

LVAD can be used as a bridge to decision in patients initially ineligible for transplant listing due to co-morbidities (i.e., renal insufficiency, increased pulmonary vascular resistances), which can potentially fully or partially resolve following a prolonged period of LVAD support, rendering, eventually, some of them eligible for heart transplantation.

Bridge to recovery is used in selected patients with potentially reversible etiologies of HF (e.g., myocarditis, peripartum/toxic cardiomyopathy) or in selected patients with advanced chronic heart failure (see Section 6).

## 4. Patient Selection

A detailed patient assessment is mandatory prior to LVAD implantation. HF status and treatment decisions will be determined by the patient’s clinical evaluation, laboratory tests, imaging studies, cardiopulmonary stress testing, and right heart catheterization.

According to the 2021 ESC guidelines for the treatment of acute and chronic HF, patients potentially eligible for LVAD implantation are those with severe symptoms despite optimal medical and device therapy, absence of major contraindications and more than one of the following: (a) LVEF <25% and if measured peakVO2 <12 mL/kg/min (and/or <50% predicted value), (b) ≥3 unprovoked HF hospitalizations in the last 12 months, (c) inotrope or temporary MCS dependence, and (d) progressive end-organ dysfunction due to low perfusion [pulmonary capillary wedge pressure (PCWP) ≥20 mmHg and systolic arterial pressure (SAP) ≤90 mmHg or cardiac index (CI) ≤2 L/min/m^2^)] [2]. The absence of severe, irreversible right ventricular dysfunction is an important prerequisite for isolated LVAD implantation.

Selecting patients for LVAD therapy is a challenging task, as successful therapy depends on strategic implantation timing and the selection of the appropriate candidate. Patients must have sufficient disease severity in order to derive a benefit, whereas patients at very late or early stages of the disease should be excluded since they are not expected to derive improvement in their clinical status and long-term survival with MCS. Taking these into consideration, the contribution of INTERMACS classification was essential in the evolving field of MCS.

**Figure 3:**
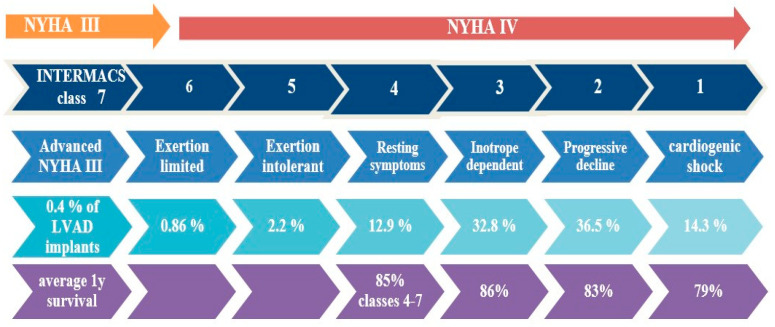
NYHA class and clinical status in INTERMACS classification. Percentage of patients implanted with a CF-LVAD and average 1-year survival, according to INTERMACS patient profile. Data from the Society of Thoracic Surgeons INTERMACS database annual report [15].

This classification was created to predict the outcome of patients receiving MCS based on their clinical and hemodynamic status. It has been proven to be a valuable tool for determining candidate appropriateness and urgency for MCS, and it is considered more specific than the NYHA classification. Briefly, patients in INTERMACS class 1 are in cardiogenic shock (crash and burn), class 2 patients are deteriorating despite inotropic therapy (sliding on inotropes), class 3 are stable on inotropes, while classes 4–7 refer to ambulatory patients with varying severity of symptoms without inotropic therapy. The INTERMACS classification also includes modifiers for profiles based on arrhythmia, frequent hospital admissions (“frequent flyers”), and temporary MCS, increasing awareness for these patients with high mortality risk [40] (Figure 3).

The outcome of patients with INTERMACS class 1–2 is worse compared to other classes after LVAD implantation. Profile 1–2 patients had the lowest survival at 30 days and 1 year and greater lengths of hospitalization compared to the rest of the profiles. These patients may benefit from short-term circulatory support prior to implantation of a durable LVAD in order to improve clinical status. INTERMACS class 3 patients have better outcomes than classes 1–2, and they are currently considered to have the optimal profile for LVAD implantation. Outcomes in profiles 4–7 following LVAD implantation are less studied, and they constitute a “grey zone”. Evidence exists that there is improvement in clinical outcomes of ambulatory chronic heart failure patients after LVAD therapy [15,41,42]. The ROADMAP trial was a prospective, nonrandomized, observational study comparing LVAD implantation vs. optimal medical therapy in patients with INTERMACS profiles 4–7. Patients in the LVAD group had improved functional status and quality of life 2 years after implantation, but they revealed a higher risk for complications compared to the optimal medical therapy group. There was not a statistically significant difference in survival between the two arms of the study [43]. Stratifying patients with INTERMACS profile, in a further ROADMAP analysis, LVAD with current technology may be a reasonable therapeutic approach in selected INTERMACS 4 patients with respect to survival and health-related quality of life despite more frequent adverse events but seems to be inappropriate for most INTERMACS 5–7 patients [44]. The MedaMACS registry enrolled 161 ambulatory AHF patients (INTERMACS profiles 4–7) on optimal medical therapy and compared outcomes with matched LVAD recipients from the INTERMACS registry. In the 2-year outcomes, 19% of patients were transplanted, 11% received LVAD therapy, and only 53% survived conservative treatment, highlighting a high-risk group. There was no difference in the intention to treat survival rates between the two groups when including less sick profiles 6–7, but there was a survival benefit with LVAD support in patients with profiles 4 and 5 compared to medical therapy [45].

In conclusion, LVAD implantation should be considered in selected patients with INTERMACS profiles 1–2, in all eligible profile three patients [6,29], while more data are needed for the benefit of relatively early LVAD support in non-inotrope dependent patients with profiles 4–7.

## 5. Referral of Patients to Advanced Heart Failure Centers

Early referral for assessment to a specialized ADHF center is very important for patient outcomes. The evaluation should be performed by a qualified and experienced Heart Team in collaboration with physicians from other specialties. Shared decision-making following a multidisciplinary work-up, including VAD and Transplant coordinators, social workers, psychologists, nutritionists, and physiotherapists, is essential for determining eligibility and timing of LVAD implantation [46]. Identifying the patient with early advanced heart failure is not an easy task. It has been suggested that patients who have marked limitations on exertion (NYHA III) despite guideline-directed optimal medical therapy should be considered for discussion with an HF specialist [29]. Recently, the American College of Cardiology, in an expert consensus document, has summarized in the acronym “I NEED HELP” high-risk features that should trigger consideration for referral for advanced HF consultation [47] (Table 2).

## 6. Considerations for Candidate Selection and Pre- and Post-LVAD Patient Management

INTERMACS classification alone is not sufficient for the selection of eligible LVAD candidates because it lacks specificity and does not take into consideration relevant co-morbidities and end-organ dysfunction. We should also consider psychosocial factors and operative risk.

### 6.1. Right Ventricular Failure (RVF)

RVF is defined by INTERMACS as documented elevations in central venous pressure (CVP) and its manifestations (edema, ascites, renal/hepatic dysfunction). RVF following LVAD implantation is considered severe when there is a need for prolonged inotropic support or RV mechanical support [48,49]. RVF complicates 10–40% of LVAD implants and is associated with multiorgan failure, longer hospitalizations, and high morbidity and mortality post-implant [50]. Therefore, simultaneous left and right ventricular assist device (BiVAD), rather than LVAD support, should be considered for bridge to transplant in patients with irreversible biventricular failure or at high risk for developing right ventricular failure after LVAD implantation, given that delayed right ventricular support after initial LVAD placement is associated with worse prognosis [15]. Consequently, identification of patients at high risk for RVF is essential, but reliable prediction of post-operative RV failure remains challenging despite the development of risk scores incorporating several variables [51]. The assessment is based on the contemporary evaluation of biochemical, hemodynamic, and echocardiographic parameters that were found to predict post-implant RVF. Biochemical parameters to be considered as high risk for the occurrence of RVF post-LVAD implantation are: Total Bilirubin ≥2 mg/dL, aspartate aminotransferase (AST) ≥80 iu/L, Creatinine ≥2 mg/dL, albumin ≤3 gr/dL, elevated INR and NT-proBNP levels [52,53,54]. With regard to hemodynamic parameters, central venous pressure (CVP) >15 mmHg, a CVP to pulmonary capillary wedge pressure ratio (CVP/PCWP) >0.6, right ventricular stroke work index (RVSWi) <300 mmHgxml/m^2^ and pulmonary artery pulsatility index (PAPi) <2 have been identified as predictors ofhigher risk for post-operative RVF [55,56,57]. Imaging plays a key role in the multiparametric pre-LVAD patient evaluation, but it is limited by complex RV geometry and load dependence. Echocardiographic parameters used in order to predict post-LVAD RVF are: TAPSE < 7.5 mm (highly specific but with poor sensitivity) [58], RV-to-LV end-diastolic diameter ratios (RV/LV ≥ 0.75) [59], fractional area change (FAC < 35%) [60], and RV free wall longitudinal strain (RVFWLS) with very promising prognostic significance (RVFWLS < −11% in absolute values) [61,62]. Patients with evidence of RV dysfunction should be hospitalized pre-operatively for aggressive management with diuretics, inotropes (dobutamine, milrinone), or temporary percutaneous MCS and then reassessed, aiming for patient optimization [31]. Evidence exists that prolonged intra-aortic balloon pump (IABP) support of patients presenting with ADHF and RV dysfunction may improve right ventricular and end-organ function [63,64]. In our clinical practice, we have observed that patients with biventricular failure demonstrate RV function improvement after prolonged IABP support, allowing safer LVAD implantation in terms of risk for post-operative RVF [65].

### 6.2. Post-Operative Temporary Mechanical RV Support

In a recent study based on an INTERMACS analysis of 6632 LVAD patients, it emerged that more than one-third of patients post-LVAD implantation had temporary or durable RV mechanical support. Severe RV failure post-LVAD was associated with poor outcomes [66]. Determining which LVAD patient requires mechanical RV support remains challenging, and the decision should be the result of a multidisciplinary team approach, taking into consideration detailed clinical, advanced echocardiographic, and hemodynamic assessment. Due to the complexity and multiple factors involved, it has been proposed that artificial intelligence (AI) technology might be able to predict the risk of right ventricular failure post-LVAD implantation more accurately [67].

The current options for temporary mechanical cardiac support are peripheral Extracorporeal Membrane Oxygenation (ECMO), IMPELLA RP, Tandem RVAD, and Protek Duo cannula. Intra-aortic balloon pump (IABP) has been used in the past to support indirectly RV perfusion with insufficient outcome results; however, modern mechanical devices have radically changed the strategic plan due to greater and more efficient RV support provided, reducing significantly the current use of IABP in acute RV failure post-LVAD implantation.

IMPELLA RP (Abiomed, Danvers) is a percutaneous microaxial pump (22 F size) positioned through the femoral vein with a distal tip to the pulmonary artery (PA) draining blood from the right atrium (RA) to PA (direct RV bypass). It has been previously shown from the the case series that IMPELLA RP is safe and efficient in improving hemodynamics and survival [68]. Tandem Protek Duo (Cardiac Assist Inc., Pittsburgh, PA 15238, USA) is a dual-lumen cannula (usually 29/31 F size) positioned percutaneously through the internal jugular vein to PA, draining blood from RA via an external centrifugal pump to PA (direct RV bypass). This percutaneous mechanical device has been proven to be feasible, relatively safe, and potentially effective with the advantage of full mobilization [69]. Veno-arterial (VA) ECMO is another valid low-cost option for temporary right ventricular support (indirect RV bypass) since it can unload RV successfully and provide adequate tissue perfusion at the same time. VA-ECMO is inserted percutaneously in the majority of cases via the femoral approach, but it might require a surgical cut approach. An inflow cannula (21–25 F size) is positioned through the femoral vein at the level of inferior vena cava/RA, draining blood via a centrifugal pump through an oxygenator and returning oxygenated blood via a retrograde outflow cannula (17–21 F size) at the femoral artery with an additional connected ante-grade-flow arterial cannula (6–8 F size) to keep limb perfusion. Other VA-ECMO configurations, such as trans-femoral/trans-axillary-pulmonary artery cannulation, might provide early mobilization and ambulation and should always be considered [70]. VA-ECMO offers the advantage of oxygenator support and should be the gold–standard approach if there is moderate to severe pulmonary hypertension and/or impaired blood gas exchange due to respiratory alterations. However, VA-ECMO increases afterload and might affect LVAD output if not managed with echocardiographic and hemodynamic monitoring to maintain adequate total cardiac output for tissue perfusion and LV unloading to avoid pulmonary edema and cardiac and pulmonary vein thrombosis.

VA-ECMO or RV assist device (RVAD) (CentriMag R^©^, Abbott, Abbott Park, IL, USA) with/without oxygenator remains the most durable approved mechanical assist device (~30 days), which in most cases is sufficient to provide RV recovery (approximately 60% weaning rate). However, long-term support might be required in cases of RV failure persistence, and temporary mechanical support should be replaced by long-term support devices such as a 2ndHeartMate 3 RV positioned (off-label use) or a paracorporeal pulsatile ventricular assist device as a biventricular support configuration (Dual Berlin Heart EXCOR) or total artificial heart (SynCardia or Aeson). These are challenging options, particularly due to the presence of LVAD support and anatomical peculiarities that determine its feasibility and applicability in these patients.

### 6.3. Aortic Insufficiency (AI)

More than mild AI is a contraindication for LVAD implantation as it creates a closed circuit that does not contribute to peripheral perfusion and, at the same time, diminishes ventricular unloading. LVAD support creates a pressure gradient across the aortic valve, which restricts its motion, leading in some cases and especially in patients with bioprosthesis to the fusion of the commissures and degeneration, resulting in worsening AI [71,72]. LVAD speed optimization in order to maintain intermittent aortic valve opening seems to be protective [73]. AI should be addressed at the time of implantation either by replacing it with a bioprosthetic valve or by approximating the aortic valve leaflets using Park’s Stich. Mechanical aortic valve replacement is contraindicated at the time of LVAD implantation [74,75].

### 6.4. Renal Dysfunction

Severe irreversible end-organ dysfunction and systemic diseases, which limit survival to <2 years, are contraindications for LVAD implantation [31,75]. Renal impairment in patients with ADHF is related mainly to poor renal perfusion, venous congestion, non-hemodynamic factors such as renin angiotensin aldosterone system (RAAS) and sympathetic nervous system (SNS) activation, inflammation, endothelial dysfunction, and anemia and to a lesser degree to intrinsic parenchymal disease from chronic co-morbidities [76]. In many patients, renal dysfunction is reversible with LVAD support, possibly due to improved cardiac output, decongestion, and decreased neurohormonal and immune-inflammatory activation. Negative predictors of improved renal function include small kidney size on ultrasonography (<10 cm), older age, and use of angiotensin-converting enzyme inhibitors [77]. Lower hemoglobin and increased proteinuria at baseline may also predict worse renal outcomes postoperatively [78]. End-stage renal failure on renal replacement therapy is an absolute contraindication for LVAD implantation because it is associated with a high risk of morbidity and mortality [79]. However, the role of LVAD therapy on renal function is an area of ongoing research [80].

### 6.5. Bleeding Risk

Bleeding is one of the most common adverse events and causes of rehospitalization in patients supported with LVADs. It manifests as surgical bleeding in the early post-operative phase and as gastrointestinal bleeding after the first 3 months of support [12,81]. Upper gastrointestinal tract is the most common site of bleeding and is typically associated with the development of arterial-venous malformations, which is thought to be a consequence of diminished pulsatility [82]. Patients should have upper and lower endoscopy, and lesions predisposing to bleeding should be treated before LVAD implantation. In order to advert pump thrombosis, patients after LVAD implantation must be treated with a coumarin anticoagulant (targeting an INR between 2–3) and an antiplatelet agent (usually aspirin 81–325 mg daily). The intensive anti-thrombotic regimen and acquired von Willebrand factor (vWf) deficiency from pathologic shear stress (including vWf unfolding and proteolysis of large into smaller, less hemostatic, multimers) are both contributing to the high prevalence of bleeding among LVAD recipients [83]. Consequently, the presence of hemorrhagic diathesis is a contraindication to LVAD implant unless coagulopathy is caused by reversible hepatic dysfunction. Low platelet count before implantation also predicts poor outcomes, and sometimes, the presence of heparin-induced thrombocytopenia antibodies needs to be excluded [75,84,85]. In the recent ARIES-HM3 trial, the exclusion of aspirin from the standard anti-thrombotic regimen was safe and reduced bleeding events, a result that may change current clinical practice [86].

### 6.6. Infection

Active infection and sepsis are contraindications to LVAD implantation. These patients should be aggressively treated with the contribution of infectious disease experts. Infections are associated with an increased risk of mortality, and they are extremely difficult to eradicate once the LVAD is inoculated, so preoperative antibiotic prophylaxis should be implemented [75,87,88,89]. With the implementation of contemporary effective anti-viral regimens, LVAD implantation seems to be a feasible treatment for selected patients with controlled HIV, HBV or HCV infection [90,91].

### 6.7. Psychosocial Evaluation

Candidates for durable MCS must be motivated, well-informed, and able to comply with the complex treatment. Additionally, they must be capable of learning the device’s operation, alarm response, and daily wound care. There is also a need to have a caregiver, usually someone from their family environment. In accordance with transplantation recommendations, all candidates for LVAD therapy should be evaluated by mental health professionals and social workers to ascertain that they are able to achieve adequate care in the outpatient setting before the decision to proceed with implantation is made [2,75,92]. Active alcohol/substance abuse, severe cognitive-behavioral disabilities or dementia, history of noncompliance, insufficient social support, and mental retardation are related to poor outcomes, and they may be contraindications to LVAD therapy [31,75,93].

### 6.8. Exercise Training (ET)

Despite the significant hemodynamic improvement and peripheral muscle strength beneficial effects [94,95], exercise capacity often remains below 50% of predicted peakVO2, with significant chronotropic incompetence similar to advanced HF [96], while 20–30% of VAD patients do not functionally recover after durable mechanical support. Exercise might, therefore, provide additional benefits to this category of patients. Exercise training appears to be feasible and safe and has been recently recommended for patients supported with LVAD [97,98,99]. Dynamic resistance and respiratory training are indicated, but they should be carefully implemented in a cardiac rehabilitation program [99,100]. ET tends to improve exercise capacity and quality of life in LVAD recipients. It might also promote myocardial recovery due to its direct effects on myocardial metabolism, as indicated by training-induced up-regulation of physiological growth signaling pathways [101]. Although the existing evidence from small trials is encouraging, further research is required in this new fascinating area. To this end, the European prospective, randomized Ex-VAD trial will assess the potential incremental beneficial effects of a supervised aerobic endurance and resistance ET program on functional capacity and quality of life in patients with LVAD [102].

### 6.9. Myocardial Recovery

Unloading of the heart during LVAD support may allow reverse cardiac remodeling and improvement in myocardial structure and function to the level that the device could be removed [103,104,105]. A study from the Harefield program in the UK showed that survival rates of patients post LVAD weaning due to myocardial recovery were similar to the post-heart transplantation outcomes [106], while another study has concluded that post-explant exercise capacity was almost similar to that of healthy controls [107]. As indicated in Table 3, sufficient recovery to allow device explantation has been observed in 3.3–73% of LVAD recipients, depending on the patient selection criteria and the specific population studied. Recent data from the U.S. multicenter RESTAGE-HF trial demonstrated that in selected heart failure patients (i.e., HF duration less than 5 years, non-ischemic cardiomyopathy, <60 yo) the implementation of standard pharmacological therapy, pump speed optimization, and monitoring of heart function can lead to LVAD explantation rates of 50% [108] (Table 3).

The Interagency Registry for Mechanically Assisted Circulatory Support (INTERMACS) provides strong evidence of what is the incidence of post-LVAD myocardial recovery in the non-selected patient populations, i.e., “all comers”. In two INTERMACS studies (performed by the Columbia group and the Utah group, respectively), approximately 13% of more than 7000 LVAD patients (that underwent post-LVAD serial echocardiograms) experienced a post-LVAD LVEF>40% [109,110].On top of these two reports from INTERMACS, a recent prospective multicenter study also investigated the reverse cardiac remodeling and recovery taking place after continuous flow LVAD [111]. The study evaluated the degree of structural (LV internal dimension at end-diastole [LVIDd]) and functional (LV ejection fraction [LVEF]) change after LVAD. Patients experiencing an improvement in LVEF ≥40% and LVIDd ≤6.0 cm were termed responders, absolute change in LVEF of ≥5% and LVEF <40% were termed partial responders, and the remaining patients with no significant improvement in LVEF were termed non responders. Among 358 LVAD patients, 34 (10%) were responders, 112 (31%) were partial responders, and the remaining 212 (59%) were non responders. The median change in LVEF was 27%, 9%, and −2%, respectively. The use of guideline-directed medical therapy for heart failure was higher in partial responders and responders. Structural changes (LVIDd) followed a different pattern with significant improvements even in patients who had minimal LVEF improvement. Altogether, the conclusion from these studies and the studies included in Table 3 is that reverse cardiac remodeling associated with durable LVAD support is not an “all-or-none” phenomenon (like pregnancy or death) and manifests in a continuous spectrum.

Predictors of cardiac recovery during LVAD support are younger age, non-ischemic HF etiology, duration of HF history less than 5 years, and less baseline left ventricular dilatation [108,109,110,111,112,113]. Additionally, consideration of novel echocardiographic markers like rotational mechanics and inflammation biomarkers could further enhance the ability to recognize patients with a higher probability of myocardial recovery before LVAD implantation [114,115]. Specific therapeutic and monitoring protocols should be implemented in these patients with a high likelihood of reverse remodeling [108,113].

In the case of myocardial recovery, LVADs can be explanted by either full removal of the pump or by deactivation and leaving different degrees of device material in the patient (decommissioning). Various techniques for apical closure of the left ventricle following the inflow cannula have been described. Occlusion of the apex with a custom-made plug with preservation of the sewing ring potentially provides the advantage of off-pump removal and the facilitation of LVAD re-implantation if heart failure reoccurs [116]. Others have suggested a ventriculoplasty technique following the removal of the sewing ring [117]. LVAD decommissioning provides the advantage of avoiding a complex redo sternotomy and has emerged as an alternative technique for LVAD explantation. The device is left in place, and the flow through it is interrupted either by ligation of the outflow graft through a small thoracotomy [118] or percutaneously by placing an AMPLATZER Vascular Plug II in the outflow graft [119]. In the case of LVAD decommissioning, the risk of infection requires close patient monitoring. Temporal use of anticoagulants and antiplatelet therapy have been described. Future studies will provide more information on the surgical technique for LVAD removal following myocardial recovery.

LVAD population is a unique myocardial recovery investigational model that has significant research implications and could be utilized to uncover novel therapeutic biology targets in order to impact the entire HF population [105].

**Table 3:** List of prospective studies investigating myocardial recovery during chronic LVAD support. Modified from Drakos et al. JACC Basic TranslSci 2017 [105].

Group, Year (Ref. #)	*n*	HF Etiology	Adjuvant Drug Therapy Protocol	Heart Function Therapy Protocol	LVAD Support Duration (Months)	Cardiac Recovery	Freedom from HF Recurrence After Explantation, Follow-Up Duration
US multicentre study, 2020 [108]	40	NICM 100%	Yes	Yes	Up to 18	50%	90% and 77%, 1 and 3 yrs respectively
US LVAD Working Group, 2007 [120]	67	NICM: 55%, ICM: 45%	Not standardized	Yes	4.5	NICM: 13.5% ICM: 3.3%	100%, 6 months
Berlin, 2008 and 2010 [121,122]	188	NICM: 100%	Not standardized	Yes	4	NICM: 19%	74% and 66%, 3 and 5 yrs, respectively
Utah Cardiac Recovery Program, 2016 [123]	154	NICM: 60%, ICM: 40%	Not standardized	Yesu	6	NICM: 21% ICM: 5%	N/A
Montefiore, 2013 [124]	21	NICM: 62%, ICM: 38%	Yes	Yes	9	NICM: 23% ICM: 0%	100%, 57 months
Gothenburg, 2006 [125]	18	NICM: 83%, ICM: 17%	Not standardized	Yes	7	NICM: 17% ICM: 0%	33%, 8 yrs
Vancouver, 2011 [126]	17	Not reported	Not standardized	Yes	7	NICM and ICM: 23%	100%, 2 yrs
Pittsburgh, 2003 [127]	18	NICM: 72%, ICM: 28%	Not standardized	Yes	8	NICM: 38% ICM: 20%	67%, 16.5 months
Texas Heart Institute, 2003 [128]	16	NICM: 75%, ICM: 25%	Yes	Yes	8	NICM: 58% ICM: 50%	78%, 14.3 months
US IMAC, 2012 [129]	14	NICM: 100%	Not standardized	Yes	3.5	NICM: 67%	87.5%, 17.5 months
Harefield, 2006 [103]	15	NICM: 100%	Yes	Yes	11	NICM: 73%	100% and 89%, 1 and 4 yrs, respectively
Harefield, 2011 [113]	20	NICM: 100%	Yes	Yes	9	NICM: 60%	83%, 3 yrs
University of Athens, 2007 [130]	8	NICM: 100%	Yes	Yes	7	NICM: 50%	100%, 2yrs

### 6.10. Complications after LVAD Implantation

The majority of LVAD recipients experience rehospitalizations for various causes, starting early after implantation. After the first year, extended survival is heavily constrained by the occurrence of adverse events and post-operative end-organ dysfunction. Infections, bleeding, stroke, and multisystem organ failure are among the most common LVAD complications and contribute to more than 50% of deaths. Furthermore, balancing the coexisting risks of bleeding and thrombosis is frequently a challenging task [131].

Infections in patients with an LVAD constitute the Achilles Heel of this therapy. Infections can be specific to the LVAD involving the driveline, the pocket, the cannulas, and the pump. Furthermore, infections can be related to LVAD (i.e., mediastinitis), and additionally, they can be non-LVAD related (i.e., pneumonia). Driveline infections are the most common device complication. Various risk factors have been identified for the occurrence of infections in patients supported with LVADs, including diabetes mellitus, obesity, and older age [132]. The most common microorganisms identified in infected patients with LVADs are staphylococcus aureus and coagulase-negative staphylococci, with lower occurrence rates for pseudomonas and fungi.

The management of the infected patient with LVAD depends on the location of the infection, the causative microorganism, and the severity, with most of the superficial driveline infections to be managed successfully with oral antibiotics for 10 days [133]. However, for those patients with more severe infections involving abscesses, it is necessary to apply an extended duration of antibiotic administration. In resistant cases, it is unavoidable to proceed to surgical management of the infection with repositioning of the driveline, or for more persistent cases, device removal or exchange. The reoccurrence of the infections, even in the cases of device removal and exchange, is high. An increase in urgency status can shorten the time for HTx for patients who face severe LVAD infections. These patients may reveal severe infections postoperatively, although post-HTx survival does not appear to be affected [134].

## 7. Conclusions

Within a relatively short period of time, technological advancements along with improved medical management have contributed to remarkable progress in the field of MCS.LVADs are undoubtedly an established therapy, saving the lives of thousands of patients suffering from end-stage HF. Outcomes are continuously improving and the number of LVAD implantations for destination therapy is consistently growing. Currently, the candidate selection process is moving toward support for ambulatory patients. However, the risk for serious adverse events is significant and remains an important limiting factor for LVAD therapy. We anticipate that innovative engineering will overcome these challenges in the near future. Smaller devices, less invasive techniques, improved biocompatibility, and elimination of the driveline are eventually expected to make LVADs available to more patients and at earlier heart failure stages.

## Figures and Tables

**Table 1:** Updated HFA-ESC criteria for defining ADHF. From M.G. Crespo-Leiro et al. Eur J Heart Fail.2018 [6], with permission.

All the following criteria must be present despite optimal guideline-directed treatment:
1. Severe and persistent symptoms of heart failure [NYHA class III (advanced) or IV].
2. Severe cardiac dysfunction defined by a reduced LVEF ≤30%, isolated RV failure (e.g., ARVC) or non-operable severe valve abnormalities or congenital abnormalities, or persistently high (or increasing) BNP or NT-proBNP values and data of severe diastolic dysfunction or LV structural abnormalities according to the ESC definition of HFpEF and HFmrEF.
3. Episodes of pulmonary or systemic congestion requiring high-dose intravenous diuretics (or diuretic combinations) or episodes of low output requiring inotropes or vasoactive drugs or malignant arrhythmias causing >1 unplannedvisit or hospitalization in the last 12 months.
4. Severe impairment of exercise capacity with Inability to exercise or low 6MWD (<300 m) or pVO2 (<12–14 mL/kg/min), estimated to be of cardiac origin.
In addition to the above, extra-cardiac organ dysfunction due to heart failure (e.g., cardiac cachexia, liver, or kidney dysfunction) or type 2 pulmonary hypertension may be present but are not required.

ARVC, arrhythmogenic right ventricular cardiomyopathy; BNP, B-type natriuretic peptide; ESC, European Society of Cardiology; HFA, Heart Failure Association; HFmrEF, heart failure with mid-range ejection fraction; HFpEF, heart failure with preserved ejection fraction; LV, left ventricular; LVEF, left ventricular ejection fraction; NT-proBNP, N-terminal pro-B-type natriuretic peptide; NYHA, New York Heart Association; pVO2, peak exercise oxygen consumption; RV, right ventricular; 6MWTD, 6 min walk test distance.

**Table 2:** I NEED HELP, useful mnemonic that should trigger consideration for referral for advanced HF consultation. From Yancy CW et al. J Am Coll Cardiol. 2018 [47], with permission.

I: Inotropes (iv)

N: NYHA IIIb-IV or persistently elevated natriuretic peptides
E: End-organ dysfunction
E: Ejection fraction ≤35%
D: Defibrillator shocks

H: Hospitalizations >1 in prior 12 months
E: Edema despite escalating diuretics
L: Low blood pressure ≤90 mmHg, high heart rate
P: Prognostic medication progressive intolerance/down-titration of guideline-directed medical therapy
